# Frequency of Successful Vaginal Birth After Cesarean Section in Women With a Previous Uterine Scar: A Cross-Sectional Study

**DOI:** 10.7759/cureus.89637

**Published:** 2025-08-08

**Authors:** Maria Rahman, Sidra Qayyum, Sara Khan, Anum Shoukat, Amina Shamim, Hamza Javed

**Affiliations:** 1 Obstetrics and Gynaecology, Ayub Teaching Hospital, Abbottabad, PAK; 2 Cardiology, Benazir Bhutto Shaheed Teaching Hospital, Abbottabad, PAK; 3 Obstetrics and Gynecology, Cumberland Hospital, North Cumbria Integrated Care NHS Foundation Trust (NCIC), Whitehaven, GBR; 4 Obstetrics and Gynecology, Combined Military Hospital, Quetta, PAK; 5 Obstetrics and Gynecology, Good Hope Hospital University of Birmingham Medical School, Birmingham, GBR; 6 Radiology, Ayub Teaching Hospital, Abbottabad, PAK

**Keywords:** adverse pregnancy: fetal outcomes, cesarean section (cs), maternal outcomes, trial of labor after cesarean section (tolac), vaginal birth after cesarean section (vbac)

## Abstract

Background: The rising rates of cesarean sections (CS) globally have increased the number of women with prior cesarean deliveries who may be candidates for vaginal birth after cesarean (VBAC). This study explores the factors influencing the success of VBAC in women with a previous CS and fetomaternal complications associated with VBAC failure.

Methods: This cross-sectional study was conducted at the obstetrics unit of Ayub Teaching Hospital, Abbottabad, Pakistan, enrolling 102 eligible women who had previously undergone a CS and were offered the option of attempting a vaginal delivery. Demographic, clinical, and obstetric data were collected on a predesigned proforma. The success of VBAC was defined as a vaginal delivery without the need for an emergency cesarean section. Statistical analysis was done using IBM SPSS Statistics software, version 27.0 (IBM Corp., Armonk, NY).

Results: Participants had a mean age of 30.36 ± 2.07 years and a mean body mass index (BMI) of 25.71 ± 0.90 kg/m². The overall VBAC success rate was 79.4% (n=81), with a failure rate of 20.6% (n=21). Maternal complications, including infection and the need for blood transfusion, were significantly higher in the VBAC failure group. Fetal outcomes also showed that VBAC failures were more likely to result in an Appearance, Pulse, Grimace, Activity, and Respiration (APGAR) score <7 at 10 minutes (P=0.006). Similarly, maternal complications were significantly higher in VBAC failures (5/21, 23.8%) than successes (3/81, 3.7%; *P*=0.009). On regression analysis, VBAC failure was found to be an independent predictor of both fetal complications (with an odds ratio of 14.610 (95% CI: 1.290-165.421, p = 0.030)) and maternal complications (with an odds ratio of 8.767 (95% CI: 1.762-44.535, p = 0.009)).

Conclusions: VBAC is a safe and effective option for women with a prior CS, with a high success rate; however, VBAC failure was found to be an independent predictor of both fetal and maternal complications. Our findings suggest the importance of a personalized approach to VBAC assessment and counselling to optimize outcomes for women undergoing trial of labor after cesarean (TOLAC).

## Introduction

The rising global rates of cesarean sections (CS) have prompted extensive discussions about their implications for maternal and neonatal health [1]. While CS remains a vital intervention for saving lives and preventing complications during childbirth, its widespread use, particularly in the absence of clear medical indications, has raised significant concerns [2]. In countries like Pakistan, for example, CS rates have surged from 3.2% in 1990 to 20% by 2018, and this trend is observed worldwide, even in developed nations with advanced healthcare systems [2-5]. The WHO has consistently emphasized that the ideal CS rate should not exceed 15%, as higher rates do not correlate with improved maternal or neonatal outcomes [6].

One of the primary drivers of the increasing CS rate is the growing prevalence of women with previous cesarean deliveries. For these women, the decision between opting for a repeat cesarean section or attempting a vaginal birth after cesarean (VBAC) is crucial. VBAC offers significant benefits, such as a reduced risk of complications associated with major abdominal surgery, including blood loss, infection, and future pregnancy complications like placenta previa and accreta [7, 8]. However, concerns about the safety of VBAC, particularly regarding the risk of uterine rupture, have led to a decline in its uptake in many settings [1]. CS is linked to immediate risks like uterine rupture, preterm birth, and stillbirth, while also influencing neonatal physiology through altered immune and gut microbiome development. Long-term effects, though less studied, suggest potential links between CS and childhood obesity, asthma, and cognitive outcomes, underscoring the need for further research on optimizing CS use for better maternal and child health [7, 8].

The choice between VBAC and repeat CS is influenced by multiple factors. Clinical predictors, including maternal age, body mass index (BMI), and the indication for the prior cesarean delivery, play an essential role in determining the likelihood of a successful VBAC [9]. Previous studies have shown that factors such as the presence of previous vaginal deliveries, a favorable Bishop score, and spontaneous labor onset significantly enhance VBAC success [10]. Moreover, recent advancements in ultrasound technology, including measurements of the lower uterine segment thickness, have provided clinicians with valuable tools for more accurately predicting uterine rupture risks and thereby aiding the decision-making process for VBAC [11].

Despite these advances, a significant gap remains in understanding the full range of factors that contribute to VBAC success. For example, while some studies suggest that maternal factors such as obesity and hypertensive disorders complicate pregnancy and reduce the likelihood of VBAC success, others have shown that women with a previous vaginal birth, including those who underwent a VBAC previously, tend to have higher chances of success [7, 9]. This growing body of evidence underscores the need for a personalized approach to VBAC counseling, considering both maternal and fetal factors in order to optimize outcomes.

We aimed to explore the determinants of VBAC success, focusing on the maternal anthropometric measures, age, and parity that can aid in selecting the appropriate candidates for trial of labor. We also aimed to assess the association of failed VBAC with fetomaternal complications. We seek to provide a comprehensive overview of the factors that should be considered when counseling women with prior cesarean deliveries on the possibility of a VBAC and the associated adverse fetomaternal outcomes.

## Materials and methods

This cross-sectional study was conducted at the Obstetrics unit of Ayub Teaching Hospital, a tertiary-care teaching institution in Abbottabad, Pakistan, to evaluate the factors influencing the success of VBAC and to identify key adverse fetomaternal outcomes associated with failed VBAC. The study focused on women who were eligible for a trial of labor after cesarean (TOLAC) and sought to understand the clinical and demographic factors that might affect their likelihood of having a successful vaginal delivery. Women's eligibility for TOLAC included having one previous low-transverse cesarean, no history of uterine rupture or classical uterine incision, no contraindications to vaginal delivery (e.g., placenta previa or transverse lie), and a singleton pregnancy in cephalic presentation. Patients presenting at or after 39+0 weeks and opting for induction of labor were admitted to be induced with cervical/membrane sweep as applicable initially. It was followed by repeat induction with either a membrane sweep if they had achieved a Bishop score > 6. However, for patients with a period of gestation (POG) of 40+6 weeks, induction was done using oxytocin infusion or if the membrane sweep had failed to induce labor. All patients opting for spontaneous labor could not be admitted to be followed, so they were not included in the study. The study was approved by the institutional review board of Ayub Teaching Hospital, Abbottabad (RC-EA-2024/072), and patient confidentiality was ensured by anonymizing all collected data.

The study population consisted of 102 women who had undergone a previous cesarean delivery and who consented to VBAC. Informed written consent was obtained from the patients, including the explanation of the details of the procedure, associated risks and benefits, and alternate treatment options available for them. The protocols for obtaining consent were approved by the ethics committee. The sample size was determined with OpenEpi (Open Source Epidemiologic Statistics for Public Health, The OpenEpi Project, Atlanta, GA, USA) and using the WHO sample size formula for cross-sectional studies. The assumptions considered included a 95% CI, 8% absolute precision, and an anticipated VBAC success rate of 78.4%, taken from existing literature [12]. Women with contraindications to vaginal delivery, such as a vertical or T-incision from a prior cesarean, multiple gestations, or conditions like placenta previa or active genital herpes, were excluded. Additionally, women with a history of uterine rupture or those who had undergone more than one CS were not included.

Data for the study were collected from the patient medical record files before discharge of each patient, which are maintained by the postgraduate residents assigned to each of the patients. The medical records provided valuable demographic information, including maternal age, height, BMI, and educational level, as well as obstetric history. Key obstetric factors such as the indication for the prior cesarean, the mode of delivery in the previous pregnancy, and any complications during the current pregnancy were also extracted from the records. The current pregnancy details included gestational age, fetal presentation, and whether labor was spontaneous or induced. Neonates were assessed in the neonatal ward by a neonatologist/pediatrician, and the mothers were followed in the obstetrics postnatal unit to collect data regarding fetomaternal complications.

The primary outcome of the study was the successful completion of a vaginal delivery, defined as a vaginal birth without the need for an emergency cesarean section. Secondary outcomes included maternal and neonatal complications, such as uterine rupture, postpartum hemorrhage, blood transfusions, neonatal Apgar scores, admission to the neonatal intensive care unit (NICU), and neonatal mortality.

Data analysis involved descriptive statistics to summarize the characteristics of the study population using IBM SPSS Statistics software, version 27.0 (IBM Corp., Armonk, NY). Continuous variables, such as maternal age and BMI, were summarized using means and standard deviations after confirming normality of the data using the Shapiro-Wilk test, while categorical variables, including indications for the prior cesarean and delivery mode, were presented using frequencies and percentages. Associations were assessed between the success or failure of VBAC and fetal and maternal outcomes using appropriate statistical tests like the chi-square test and t-test, taking p-value < 0.05 as significant. A logistic regression analysis was conducted to assess the factors associated with successful VBAC and the association of failed VBAC with fetomaternal complications.

## Results

Table 1 presents the baseline maternal characteristics of the study population, expressed as mean ± standard deviation (SD). Participants had a mean age of 30.36 ± 2.07 years, with an average weight of 65.30 ± 4.64 kg and height of 1.59 ± 0.03 m, resulting in a mean BMI of 25.71 ± 0.90 kg/m². These values indicate a relatively homogeneous cohort with minimal variability in anthropometric measures.

**Table 1:** Characteristics of the study participants

Maternal characteristics	Mean ± SD
Age (years)	30.36 ± 2.07
Weight (kg)	65.30 ± 4.64
Height (m)	1.59 ± 0.03
BMI (kg/m^2^)	25.71 ± 0.90

Table 2 summarizes key clinical and demographic characteristics of participants (N=102). Most were multiparous (81, 79.4%) or uniparous (15, 14.7%), with 59.8% (n=61) being housewives. Adverse fetal outcome assessed was Appearance, Pulse, Grimace, Activity, and Respiration (APGAR) scores <7 at 10 minutes, which was recorded in five (4.9%) neonates. Maternal complications were rare (infection: two, 2.0%; transfusion: seven, 6.9%).

**Table 2:** Key clinical and demographic characteristics of participants (N=102) APGAR: Appearance, Pulse, Grimace, Activity, and Respiration; VBAC: vaginal birth after cesarean

Clinical and demographic characteristics		N (%)
Parity	Uniparous	15 (14.7%)
	Multiparous	81 (79.4%)
	Grand multiparous	6 (5.9%)
Occupation	Housewife	61 (59.8%)
	Office job	30 (29.4%)
	Outdoor job	11 (10.8%)
Fetal outcome	APGAR <7 at 10 min	5 (4.9%)
	APGAR >7 at 10 min	97 (95.1%)
Maternal outcomes	Infection	2 (2.0%)
	Normal	92 (90.2%)
	Transfusion needed	7 (6.9%)
	Uterine atony	1 (1.0%)
VBAC outcome	Failure	21 (20.6%)
	Success	81 (79.4%)

Table 3 compares maternal and fetal outcomes based on VBAC success (N=81) versus failure (N=21). Maternal complications were significantly higher in VBAC failures (5/21, 23.8%) than successes (3/81, 3.7%; P=0.009). Similarly, the only adverse fetal outcome, APGAR <7 at 10 minutes, was more frequent in failures (4/21, 19.0%) versus successes (1/81, 1.2%; P=0.006). Parity distribution showed no significant association with VBAC outcome (P=0.184).

**Table 3:** Association between fetal and maternal outcomes/characteristics and VBAC outcomes, with statistical significance (p-values). The chi-square test was applied to this data. VBAC: vaginal birth after cesarean; APGAR: Appearance, Pulse, Grimace, Activity, and Respiration

Characteristics		VBAC outcome		Total	p-value
		Failure	Success		
Maternal outcome	Complication	5	3	8	0.009
	Normal	16	78	94	
Fetal outcome	APGAR <7 at 10 minutes	4	1	5	0.006
	APGAR >7 at 10 minutes	17	80	97	
Parity	Uniparous	3	12	15	0.184
	Multiparous	15	66	81	
	Grand multiparous	3	3	6	

Table 4 presents the comparison of demographic and anthropometric characteristics, like age, weight, height, and BMI, between women who had a VBAC and those who experienced a failed attempt. Although the mean values for all variables were slightly higher in the success group compared to the failure group, none of the differences reached statistical significance. Specifically, women with successful VBAC had a slightly higher mean age (30.53 vs. 29.70 years, p = 0.104), mean weight (65.72 vs. 63.67 kg, p = 0.070), mean height (1.597 vs. 1.582 m, p = 0.102), and BMI (25.79 vs. 25.43 kg/m², p = 0.108). These findings suggest that while the successful group tended to have marginally higher anthropometric measures, these factors were not statistically significant predictors of VBAC outcome in this sample.

**Table 4:** Comparison of maternal demographic and anthropometric characteristics between successful and failed VBAC groups. Student's t-test was applied to this data. VBAC: vaginal birth after cesarean

Parameters	VBAC Outcome	N	Mean	Sstandard deviation	p-value
Age	Success	81	30.527	1.8784	0.104
	Failure	21	29.695	2.6299	
Weight (kg)	Success	81	65.721	4.3257	0.070
	Failure	21	63.667	5.4988	
Height (m)	Success	81	1.5968	.03008	0.102
	Failure	21	1.5819	.03723	
BMI (kg/m^2^)	Success	81	25.785	.8678	0.108
	Failure	21	25.429	.9717	

Table 5 presents the results of univariate and multivariate logistic regression analyses assessing factors associated with the success of VBAC. In the multivariate model, two factors were found to be associated with VBAC failure. VBAC failure significantly increased the risk of maternal complications, with an adjusted odds ratio (AOR) of 8.767 (95% CI: 1.762-44.535, p = 0.009). Similarly, VBAC failure was associated with significantly higher odds of a low APGAR score (<7 at 10 minutes), with an AOR of 14.610 (95% CI: 1.290-165.421, p = 0.030). Although age, BMI, and parity did not reach statistical significance in the adjusted analysis, they showed trends toward association in the univariate models. These findings highlight the importance of VBAC success in predicting maternal and fetal outcomes.

**Table 5:** Predictors of successful VBAC and association of fetal and maternal complications associated with failed VBAC based on univariate and multivariate logistic regression analyses. VBAC: vaginal birth after cesarean; UOR: unadjusted odds ratio; AOR: adjusted odds ratio; APGAR: Appearance, Pulse, Grimace, Activity, and Respiration

Factors	Successful VBAC		Univariate logistic regression			Multivariate logistic regression		
	Yes	No	p-value	UOR	95% CI for UOR	p-value	AOR	95% CI for AOR
Age (Mean ± SD)	30.53 ± 1.88	29.70 ± 2.63	0.104	0.816	0.683 - 1.042	0.867	1.055	0.563-1.977
BMI (Mean ± SD)	25.78 ± 0.87	25.43 ± 0.97	0.108	0.614	0.339 – 1.114	0.499	0.584	0.123– 2.775
Maternal outcomes								
Complications	3 (8)	5 (8)	0.007	8.125	1.716 – 37.489	0.009	8.767	1.762– 44.535
No complications	78 (94)	16 (94)	1			1		
Fetal outcomes								
APGAR score <7 at 10 mins	1 (5)	4 (5)	0.011	18.82	1.978 – 179.12	0.030	14.610	1.290– 165.421
APGAR score >7 at 10 mins	80 (97)	17 (97)	1			1		
Parity								
Uniparous	12 (15)	3 (15)	0.183	0.250	0.033 – 1.923	0.10	0.123	0.010– 1.499
Multiparous	66 (81)	15 (81)	0.087	0.227	0.042 – 1.239	0.078	0.175	0.025– 1.218
Grand multiparous	3 (3)	3 (3)	1			1		

## Discussion

This study aimed to assess the factors influencing the success of VBAC. The overall success rate of VBAC in this cohort was 79.4%, with a failure rate of 20.6%. This high success rate aligns with findings from several studies in the literature, which suggest that VBAC remains a highly successful option for women with a previous CS, especially when appropriately selected [13].

The mean age of participants in this study was 30.36 ± 2.07 years, with a mean BMI of 25.71 ± 0.90 kg/m². These figures reflect a cohort that is mostly in their early thirties and has a slightly overweight BMI, which is consistent with the general population of women attempting VBAC. Research by Wu et al. (2019) found that younger maternal age was associated with higher VBAC success rates, particularly when the woman's BMI was within the normal range [14]. While the average BMI in this study is slightly higher than the ideal (18.5-24.9 kg/m²), other studies indicate that women with a BMI under 30 kg/m² have favorable chances of VBAC success. However, Mekonnen and Asfaw (2023) highlight that obesity (BMI ≥ 30 kg/m²) can significantly reduce the likelihood of VBAC success, as it is associated with higher rates of labor complications [15].

In our study, 79.4% of participants were multiparous, while 14.7% were uniparous, and 5.9% were grand multiparous. Parity has long been considered an important predictor of VBAC success. However, our study found no statistically significant relationship between parity and VBAC outcomes (P=0.184), which is in contrast to some studies suggesting that multiparous women are more likely to succeed in VBAC due to their experience with vaginal deliveries. Mekonnen and Asfaw (2023) noted that women with a history of previous spontaneous vaginal delivery had significantly higher odds of successful VBAC (AOR: 2.92; 95% CI: 2.02, 4.23) [15]. Despite these findings, the current study did not observe a significant association between parity and VBAC outcome, which suggests that other factors, such as maternal health and fetal outcomes, may play a more significant role in determining VBAC success.

The study demonstrated that 95.1% of infants had normal APGAR scores, with only 4.9% of the newborns experiencing an APGAR score below six at 10 minutes. Adverse fetal outcomes, as indicated by APGAR scores, were more frequent in VBAC failures (19.0%) compared to VBAC successes (1.2%), with this difference being statistically significant (P=0.006). These findings are consistent with Charitou et al. (2019), who reported that the risk of fetal complications, such as low APGAR scores, is higher in VBAC failures [16]. Rizzo et al. (2020) also found that adverse fetal outcomes, including lower APGAR scores, were more common in women who had unsuccessful VBAC attempts, reinforcing the importance of close fetal monitoring during TOLAC [17].

Maternal complications were also more frequent in VBAC failure cases. In our study, 23.8% of women with VBAC failure experienced maternal complications such as infections and the need for blood transfusions, compared to only 3.7% in the success group (P=0.009). This higher rate of complications in VBAC failures is consistent with findings from Charitou et al. (2019), who emphasized that failed VBACs are associated with significant maternal risks, including hemorrhage and uterine rupture [16]. In their study, Charitou et al. (2019) observed that uterine rupture rates in VBAC failures ranged from 0.2% to 0.7%, with maternal mortality also being higher compared to elective CSs [16]. The maternal complications seen in our cohort align with these findings, highlighting the risks associated with VBAC failure.

In contrast, the overall maternal morbidity in VBAC successes in our study was relatively low, with only 3.7% of women experiencing complications, including minor infections and transfusions. This is consistent with Tilden et al. (2017), who found that successful VBACs were generally associated with fewer complications compared to repeat CSs [18]. VBAC, therefore, remains a safer alternative for women with a prior CS when it is successfully completed, offering a shorter recovery time and fewer risks of long-term complications such as those linked with repeated CSs.

The statistical analysis revealed that maternal complications and fetal outcomes were significantly associated with VBAC failure, but parity and maternal age were not. Wu et al. (2019) also found that factors such as labor induction, previous vaginal birth, and maternal health conditions (e.g., diabetes and hypertensive disorders) significantly affected VBAC success [14]. While parity was not significant in our study, other studies have suggested that a previous successful vaginal birth is one of the strongest predictors of VBAC success. The results from Boatin et al. (2024) further corroborate these findings, demonstrating that, in Sub-Saharan Africa, women who had a history of successful VBAC were more likely to experience success again in subsequent pregnancies [11].

This study highlights that VBAC is a generally effective and safe option for women with prior CSs, with a high success rate of 79.4%. The factors associated with VBAC failure in our sample, maternal complications and adverse fetal outcomes, are consistent with findings in the literature. The lack of a significant association with parity further suggests that other factors, such as maternal health and fetal outcomes, should be considered more critically when determining the likelihood of VBAC success. These findings underscore the importance of individualized counseling for women attempting TOLAC. VBAC, when appropriately selected and monitored, offers a safe alternative to repeat CSs, with fewer complications and a faster recovery.

The limitations of the study include the single-center nature of the study. However, a statistically significant sample size calculated with the appropriate formula and assumptions makes the study powerful.

## Conclusions

VBAC remains a clinically viable and safe option for appropriately selected women with a history of cesarean delivery, demonstrating a high success rate of 79.4% in this study. Our findings underscore that failed VBAC attempts are significantly associated with increased risks of both maternal complications and adverse fetal outcomes, particularly lower APGAR scores and the need for interventions such as transfusions.

Importantly, while demographic and anthropometric factors such as age, BMI, and parity did not independently predict VBAC success, the presence of maternal or neonatal complications was strongly correlated with VBAC failure. These results suggest that clinical decision-making around VBAC should extend beyond baseline maternal characteristics and include vigilant assessment of maternal and fetal well-being throughout the trial of labor. Moreover, close monitoring during labor and preparedness for timely surgical intervention are crucial to mitigating complications in the event of VBAC failure.
